# Effects of Citrus Fiber on the Gel Properties of Mutton Myofibrillar Protein

**DOI:** 10.3390/foods12040741

**Published:** 2023-02-08

**Authors:** Chenyan Zhu, Shouwei Wang, Yanhong Bai, Shunliang Zhang, Xin Zhang, Qianrong Wu, Xiangli He

**Affiliations:** 1 China Meat Research Center, Beijing 100068, China; 2 College of Food and Bioengineering, Zhengzhou University of Light Industry, Zhengzhou 450001, China

**Keywords:** citrus fiber, myofibrillar protein, gel properties, microstructure

## Abstract

This work investigated the effects of different additions of water-soluble citrus fiber (SCF) and water-insoluble citrus fiber (ICF) on the gel properties of the mutton myofibrillar protein (MP). The key parameters of water-holding capacity (WHC), rheological properties, and microstructure were evaluated. The addition of 2.5–10% of SCF and ICF significantly improved (*p* < 0.05) the WHC and gel strength of mutton MP gel. The rheological results showed that the viscoelasticity of MP with 5% SCF was the best, and the T_2_ relaxation time of the gel was significantly shortened. SEM results showed SCF reduced the number of pores in the MP gel, forming a more compact network structure. ICF stabilized the MP gel network structure as a filler after water absorption and expansion. However, the gel lost moisture under the action of strong external force (freeze-drying), which left large pores. These data confirmed that SCF and ICF could effectively improve the gel properties of meat products.

## 1. Introduction

Myofibrillar protein (MP) is a salt-soluble protein that contains several types of proteins (myosin, actin, actomyosin, etc.), accounting for approximately 50% of muscle protein [1,2]. During the heating process of meat products, MP molecules or other non-protein components will interact chemically or physically to form a gel network structure [3]. Ferry [4] summarized the process of heating MP to form gels into two steps: firstly, small molecular proteins are denatured and expanded by heating, folding, and polymerization to form long chains or branched chains. Secondly, long-chain macromolecules undergo cross-linking polymerization to reach the gel point, forming a stable network gel structure. The formation of the gel structure determines the texture characteristics, shelf life, and stability of meat products [5]. Therefore, studying the functional characteristics of MP in the simulation system can simulate the reaction of meat production and provide a reliable method for studying the quality of meat products.

Natural polysaccharides with high natural yield, renewability, and healthy benefits, are a promising source of functional components of meat products. The researchers are keen to add natural polysaccharides to improve the gel and texture properties of meat products [6,7,8]. Some reports have indicated that an appropriate amount of polysaccharides (especially dietary fiber) can improve the gel properties of meat products [9,10,11]. In Japan, there are specific healthy meat products made by adding dietary fiber. Moreover, other meat products containing fiber, metal elements, and plant proteins have also been approved in Japan [12]. Many polysaccharides exhibit significant rheological properties, such as thickening, stabilization, gelation, and emulsification. Some of these polysaccharides can have a large effect on the texture characteristics of foods, even at very low concentrations [13]. Ayadi et al. [14] found that 0.8% carrageenan significantly increased the hardness and cohesiveness of sausages. Zhuang et al. [15] reported that sugarcane dietary fiber significantly improved the secondary structure and gel strength of MP. Zhou et al. [16] reported that κ-carrageenan and locust bean gum significantly improved the gel strength of chicken MP.

Citrus fiber (CF) is a kind of pure natural functional food polysaccharide extracted from citrus fruits, which is rich in dietary fiber. Moreover, CF has good properties of water retention, oil retention, thickening, and emulsification, and is widely used in food, health products, and other fields [17]. The main polysaccharide components in CF are pectin and cellulose. The inherent viscosity of pectin has established the advantages of CF. As another main component of CF, hemicellulose increases its viscosity during hydration, which can improve the water-holding capacity of CF [17,18]. Studies have shown that adding an appropriate amount of CF to meat products significantly improved the WHC, and prolonged the storage life of the products [18]. However, the mechanism of interaction between CF and MP gel has not been reported, and its potential relationship is not clear.

This study aimed to provide guidance for the development and utilization of SCF and ICF in the meat industry. To this end, we studied the whiteness, WHC, gel strength, molecular forces, rheology, LF-NMR, and microstructure of mutton MP to evaluate the effects of different additions of SCF and ICF on the gel properties of mutton MP.

## 2. Materials and Methods

### 2.1. Materials

Fresh mutton hind legs were taken from a farmers’ meat market (Beijing, China). Both SCF and ICF were provided by Lemont (Xingtai, China). All the reagents used in the experiment were purchased from Hushi (Shanghai, China) and Solarbio (Beijing, China), and all of them were chemically pure.

### 2.2. Extraction of MP

The MP was prepared according to the method of Hu et al. [7]. The fresh mutton hind legs were chopped and homogenized (AM-3, Seiki Seisakusho, Tokyo, Japan) for 1 min with phosphate buffer solution (PBS, 8.1 mM Na_2_HPO_4_, 1.9 mM NaH_2_PO_4_, 0.1 M NaCl, pH 7.0, 4 °C). The mixed solution obtained from the homogenate was centrifuged at 8000 r/min for 10 min (SORVALL LYNX 4000, Thermo Fisher Scientific, Shanghai, China) and washed four times in the same way. Finally, MP was obtained by filtration and centrifugation for 15 min. The concentration of MP was assessed by the biuret method.

### 2.3. Sample Preparation

The MP solution concentration was adjusted to 40 mg/mL with PBS, and 0%, 2.5%, 5%, 7.5%, 10% SCF, and ICF were added, respectively. Then homogenized at 10,000 r/min for 30 s and centrifuged at 5000 r/min for 3 min to remove bubbles in the MP solution. Aliquots of 5 g of mixed MP solution were transferred into a 5 mL glass beaker. The gels were heated to 80 °C in a water bath and kept for 30 min, then cooled in an ice-water mixture for 30 min and stored at 4 °C.

### 2.4. Whiteness

The whiteness of the MP gel was measured by a colorimeter (CR-400/410 colorimeter, Konica Minolta, Japan). Where L* represented brightness value, a* represented redness/greenness value, and b* represented yellowness/blueness value. The whiteness was calculated using Equation (1):

(1) $$\begin{array}{c} \;\mathrm{Whiteness}=100-\sqrt{\left(100-L^{*2}\right)+a^{*2}+b^{*2}} \end{array}$$

### 2.5. Gel Strength

The strength of the protein gel refrigerated overnight was measured by a texture analyzer (Stable Micro Systems, Godalming, UK). The gels were cut into cylinders 2 cm high and 1.5 cm in diameter. P/0.5R type probe was used, pre-test speed 5 mm/s, test speed 2 mm/s, post-test 5 mm/s, trigger force 5 g, and compression distance 40%.

### 2.6. WHC

The WHC of protein gel was measured according to Sanchez-Gonzalez et al. [19]; 5 g protein gel was accurately weighed, wrapped with filter paper, and placed in a centrifuge tube. The bottom of the centrifuge tube was covered with a layer of skimmed cotton to facilitate water absorption; 5000 r/min centrifugation 10 min. WHC was the ratio of the weight of gel weight before and after centrifugation.

### 2.7. Molecular Forces

Molecular forces were measured according to Niu et al. [20]. Three denaturing solutions: 0.25% β-mercaptoethanol (β-ME), 0.5% SDS, and 8 M urea in 50 mM sodium phosphate (pH 7.0) were used to detect disulfide linkages, hydrophobic association, and hydrogen bonding, respectively. Aliquots of 2 g of MP gels were mixed with 18 mL of each denaturing solution and homogenized for 20 s with an AM-3 high-speed homogenizer at 10,000 r/min. The mixed solutions were then heated in a water bath at 80 °C for 1 h and cooled to room temperature before centrifugation at 10,000 r/min for 10 min. The MP gel solubility was the ratio of concentration before and after centrifugation.

### 2.8. Dynamic Rheological Properties

The MP concentration was diluted to 20 mg/mL, and the dynamic rheology was measured by a rheometer (Discovery DHR-2, TA instrument Co., Waters Technology, New Castle, DE, USA) according to the method of Zhang et al. [8]. A 40 mm parallel steel plate was used. Parameter setting: the frequency was 0.1 Hz, the strain was 1%, the gap was 1 mm, and it was heated from 25 °C to 90 °C at a rate of 2 °C/min. The storage modulus (G′) and loss modulus (G″) were recorded.

### 2.9. LF-NMR

The water distribution and relaxation time (T_2_) of MP gel were measured by NMI20 low-field NMR Analyzer (Niumag Electric Corp., Suzhou, China). The parameters were set referred to the method of Han et al. [21]. The temperature was set to 32.00 ± 0.01 °C, the frequency was 18 MHz, 16 scans were performed, the echos was 12,000, the repetition interval was 100 ms, and the time between 90° and 180° pulses was 200 μs.

### 2.10. Scanning Electron Microscopy (SEM)

According to the method of Shi et al. [3], the MP gel was cut into cubes about 1 × 1 × 1 cm, fixed with liquid nitrogen, and dried with an LGJ-30D lyophilizer (Beijing Sihuan Qihang Technology, Co., Ltd., Beijing, China). The dried protein gel was sprayed with gold, and the morphology of the sample was observed under 15 Kv accelerated voltage by SU8000 Series scanning electron microscope (Hitachi, Tokyo, Japan).

### 2.11. Statistical Analysis

Each experiment was repeated at least in triplicate. One-way ANOVA was processed by IBM SPSS 21.0 Statistics (IBM, Co., Ltd., North Castle, NY, USA). Significant differences between means were identified using Duncan’s multiple range tests. Principal component analysis (PCA) was performed with Origin 2021 (OriginLab, Co., Ltd., Northampton, MA, USA).

## 3. Results and Discussion

### 3.1. Whiteness

**Figure 1:**
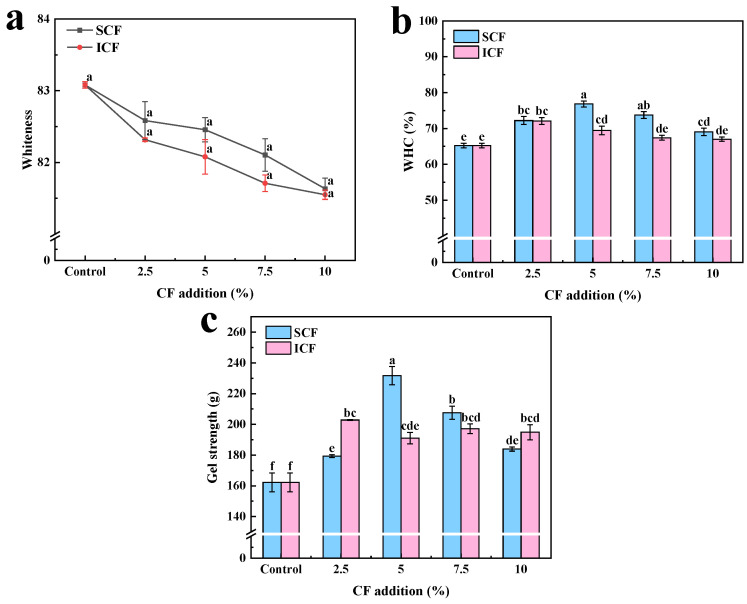
The whiteness (**a**), WHC (**b**), and gel strength (**c**) of the MP gels with different treatments. Each treatment was repeated at least in triplicate. Data are expressed as the mean ± SD. Different letters indicate significant differences (*p* < 0.05).

Whiteness is an important index that directly affects consumer preference for meat products [22]. The whiteness of the gel after adding SCF and ICF is shown in Figure 1a. The control group had the highest gel whiteness value (83.08). The whiteness of the gel had no significant effect after adding SCF and ICF. However, the whiteness of ICF was lower than SCF for the same amount of addition. This phenomenon can be attributed to the fact that ICF was darker than SCF. Leng et al. [23] found that the color of gel was determined by the color of the additives. Similarly, Kong et al. [10] found that the color of starches determined the whiteness of surimi gel. It was also possible that during the heating process, polysaccharides reacted with protein amino groups to produce melanin-like substances, leading to a darker color.

### 3.2. WHC

The WHC is closely related to the juiciness, tenderness, taste, and other qualities of meat products in the processing process [24]. The WHC of protein gel increased significantly (*p* < 0.05) after adding SCF and showed a trend of first increasing and then decreasing (Figure 1b). After adding 5% SCF, the WHC increased by 17.80% compared with the control group. This was mainly because the pectin contained in SCF could combine with water to form a hydrogel structure [25]. During the heating process, the water absorption of SCF was transformed into a sol structure with high viscosity, which made the network structure of the protein gel more compact. [10]. The change of structure was also further confirmed by the SEM. After adding ICF, the WHC of the gel was significantly improved (*p* < 0.05), but lower than SCF. Since ICF was dispersed into the protein after water swelling and played a filling role, the WHC of the protein gel increased. However, excessive ICF would compete with protein for water molecules. Under the effect of strong centrifugal force, water molecules were hard to be trapped, which resulted in the dissipation of water and the reduction in WHC.

### 3.3. Gel Strength

The gel strength of MP after adding SCF and ICF is shown in Figure 1c. Compared with the control group, the gel strength of MP gel was significantly increased (*p* < 0.05) after adding SCF and ICF. The change trend of gel strength was positively correlated with WHC, and the effect of adding SCF was better than ICF. As a water-soluble polysaccharide, SCF could interact with protein molecules to expose the sulfhydryl groups in the protein [8], thus improving the structure of the protein gel. However, with the increasing amount of SCF, the improvement of gel strength decreased significantly (*p* < 0.05). Zhou et al. [16] believed that a higher concentration of polysaccharides would destroy the polymerization and aggregation of the protein, thus affecting the gel strength. As an insoluble dietary fiber, ICF could improve the gel strength of protein gel by cross-linking with protein and embedding the gap in the protein network. Zhuang et al. [26] found similar results in the investigation of the gel strength of sugarcane dietary fiber on MP.

### 3.4. Molecular Forces

Hydrogen bonding, hydrophobic association, and disulfide linkages play critical roles in the cross-linking of proteins. Urea, SDS, and β-ME can serve as dissociation reagents to destroy the spatial structure maintained by the corresponding forces. The molecular force is reflected by the change in the solubility of the protein gel. The solubility of protein gel increased by 8.29% after adding 5% SCF (Table 1) compared to the control group, indicating that hydrogen bonding was involved in the gelation process of the protein network. The results of protein gel in 0.5% SDS and 0.25% β-ME solution were similar to those observed in 8 M urea. These results indicated that hydrophobic association and disulfide linkages were also key molecular forces for protein gel formation, and SCF can promote the formation of the hydrophobic association and disulfide linkages. It may also be that SCF promoted the expansion of the MP structure, exposing more sulfhydryl groups and hydrophobic groups [8]. According to the solubility, it was found that the contribution of the molecular force to maintain the spatial structure of the MP gel was hydrophobic association > hydrogen bonding > disulfide linkages, which was similar to the results of Niu et al. [20]. After adding ICF, the solubility of protein gels in 8 M urea, 0.5% SDS, and 0.25% β-ME solution had no significant change, indicating that ICF had no effect on maintaining most of the spatial structure of the protein gel network. In conclusion, SCF played an active role in maintaining the molecular forces of a protein gel.

### 3.5. Dynamic Rheological Measurements

**Figure 2:**
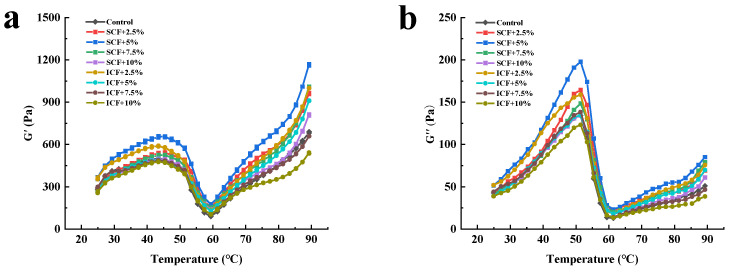
The G′ (**a**) and G″ (**b**) of the MP gels with different treatments.

Figure 2 shows the effect of different additions of SCF and ICF on the G′ and G″ of mutton MP. As indicated in Figure 2a, the value of G′ increased slowly with the increase in temperature, reaching the first peak at about 43 °C, indicating that the MP gel protein network structure has been initially formed. Jiang and Xiong [27] speculated that it was mainly caused by the unfolding and binding of the myosin head. After that, the MP gel G′ decreased rapidly and reached the lowest point at about 57 °C. It may be that the expansion and cross-linking of the myosin tails destroyed the temporary protein network structure. [28]. When the temperature was higher than 57 °C, G′ continued to increase, and myosin and actomyosin had been completely denatured, indicating that an irreversible high-viscoelastic gel structure had been formed [8].

### 3.6. LF-NMR

After adding 2.5–10% SCF, the G′ value increased at first and then decreased, which was always higher than the control group. It was shown that the SCF could improve the elasticity of gels, and the optimal condition was with 5% SCF addition. Zhuang et al. [6] believed that polysaccharides could interact with proteins to form an “interpenetrating” structure, thus affecting the structure of the gel. The addition of 2.5–5% of ICF could improve the G′ of the MP gel; when the addition amount was increased to 7.5%, the G′ of the gel was lower than the control group. Debusca et al. [29] found that insoluble dietary fiber had no effect on the thermal denaturation behavior of the protein. However, when an excessive amount of ICF was added, it would compete with protein molecules for water molecules during water swelling. As a result, fewer water molecules could be bound to the protein molecules, which prevented the cross-linking between protein molecules, reducing the G′ of the MP.

G″ could be used to characterize the protein gel viscosity characteristics. After adding SCF and ICF, the change trend of G″ was the same as that of G′. These data demonstrated that adding 2.5–10% SCF or 2.5–5% ICF could effectively improve the viscoelasticity of MP gels.

**Figure 3:**
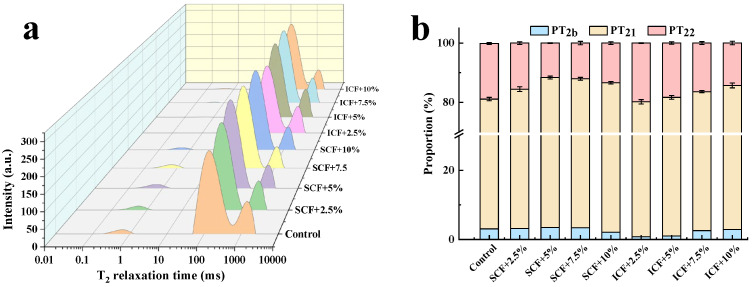
Effects of SCF and ICF on the distribution of the T_2_ relaxation times (**a**) and peak areas (**b**) of MP gels.

The distributions of T_2_ relaxation times on the MP gels with SCF and ICF is shown in Figure 3a. Zhuang et al. [15] found that the relaxation time of water was usually described by three T_2_ components: protein-associated water (T_2b_, 1–10 ms), immobilized water (T_21_, 200–400 ms), and free water (T_22_, 1000–2000 ms).

**Figure 4:**
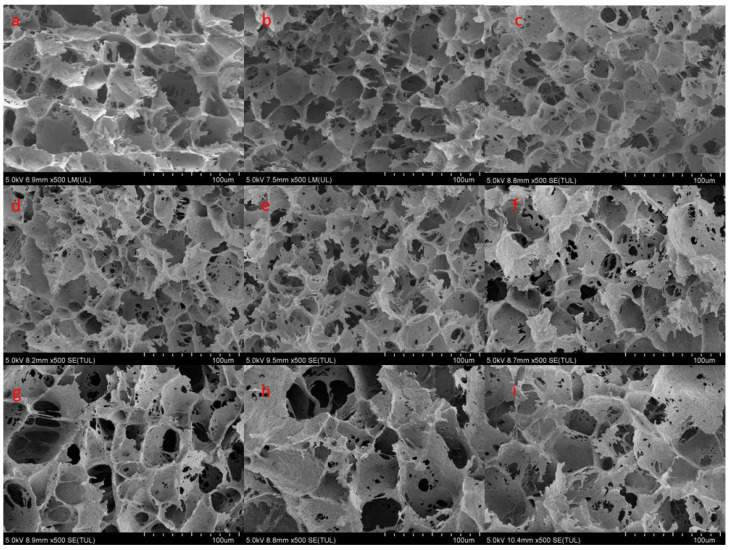
(**a**–**i**) are SEM images (×500) of MP gels with different additions of SCF and ICF. (**a**) Control group, (**b**–**e**) 2.5–10% SCF, (**f**–**i**) 2.5–10% ICF.

In the present study, the T_2_ relaxation times of MP gels after adding SCF and ICF were similar to the above ranges. The T_2_ relaxation time indicated the degree of interaction between the substance and its surrounding chemical environment. The shorter relaxation times indicated faster exchange processes and stronger interactions [21]. The results showed that after adding SCF, the peak shifted to the left, and the T_21_ relaxation time of the gel system decreased significantly (*p* < 0.05). Especially after adding 5% SCF, the T_21_ relaxation time decreased from 57.22 ms to 37.65 ms. The addition of SCF could restrict the movement of water molecules in MP gel, weaken the fluidity of water molecules, and shorten the relaxation time. This may be due to the fact that under heat induction, polysaccharides promoted protein–protein interaction and made the gel structure denser (Figure 4). A large number of water molecules were trapped inside the MP gel network structure, and the free water content was reduced. ICF had good water-locking ability after absorbing and swelling, which restricted the movement of free water to a certain extent. Under heat-induced conditions, ICF will entangle with the protein, forming a tight cavity, trapping the free water in the MP gel network structure, and allowing the free water to migrate towards the immobilized water part.

The percentage of peak area can represent the proportion of water in a certain state under different conditions and infer the migration of water [30]. The results showed that with the increase in SCF addition, the proportion of immobilized water in the MP gel gradually increased. When the addition of SCF was 5%, the immobilized water content reached the maximum, which increased by 20.00% compared with the control group. The proportion of free water decreased from 19.00% in the control group to 13.50%, indicating that the free water was converted to immobilized water. It was consistent with the results of WHC. With the increase in the amount of ICF, the proportion of immobilized water in the gel decreased gradually, but it was still higher than in the control group. It indicated that ICF could also promote the transformation of immobilized water to free water in the gel system.

### 3.7. SEM

Figure 4 shows the microstructure of the gel after adding SCF and ICF. In the control group (Figure 4a), the pores of the gel structure were larger, and the distribution was uneven. It was speculated myosin was thermally denatured, part of the water escaped, and plenty of pores were formed, which led to the loosening of the entire MP gel structure [15]. After adding SCF, the structure of the protein gel was obviously improved, the stomata became less, and the structure became uniform and compact. In particular, the addition of 5% SCF showed the best three-dimensional network structure. However, with the increase in SCF addition, the interaction between protein–protein became more intense, and the extra SCF absorbed water to form a “weak gel” structure [31], cross-linking with protein molecules, which changed the structure of the MP gel.

After the addition of ICF, the structural state of the protein gel was different from the control group and the addition of SCF. With the increase in ICF addition, the microstructure of protein gel became rougher, the pores were larger, and there were a large number of small pores on the pore wall (Figure 4f–i). It was speculated that ICF may be embedded in protein molecules after water absorption and expansion, and its structure had not undergone a chemical change. After freeze-drying, MP gels lost moisture and left a large number of small holes in the gels and fiber. Similarly, Su et al. [32] found that when citrus fiber was not completely saturated with water absorption, the fiber structure was loose, resulting in a porous structure of the fiber. In contrast, the structure of the protein gel added with SCF was more compact, and the pores were more uniform and fine. This result was consistent with the measured gel strength and LF-NMR.

### 3.8. PCA

**Figure 5:**
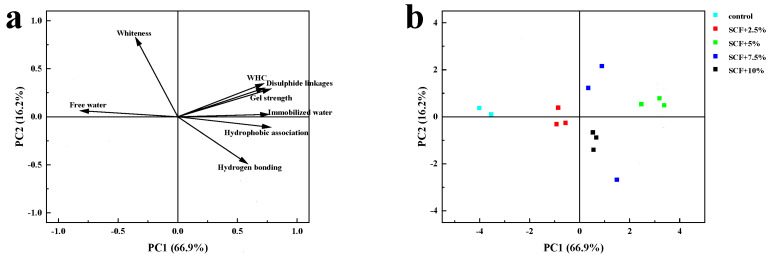
Graphical of principal component (PC) analysis of SCF and MP mixed gels of PC2 and PC1: (**a**) factor load and (**b**) factor score.

PCA is usually used to analyze the differences between samples [20]. As shown in Figure 5a, the two principal components explained 83.1% of the total variation, indicating that the correlation between the original data was strong. The first component (PC1) was positively correlated with WHC, gel strength, molecular force, and immobilized water, and negatively correlated with whiteness and free water. The second component (PC2) was positively correlated with whiteness, WHC, gel strength, disulfide bonding, and water distribution, and negatively correlated with hydrogen bonding and hydrophobic association. The main reason was that SCF could promote the structure expansion, hydrophobic association, and disulfide bonding of MP gels were exposed. The exposed groups led to the enhancement of the spatial structure of the gel, forming a dense gel structure, and the free water was transformed into immobilized water.

Figure 5b shows the quadrant position of the control group and the treatment groups on the score chart. The control group was located in the second quadrant, and the treatment groups moved to the upper right with the increase in the amount of SCF, indicating that the treatment groups had a significant effect on the PC1. Treated with 5% SCF was farthest from the control group, indicating that 5% SCF had the best effect on the improvement of a protein gel. Therefore, according to the results of PCA, the treatment groups had significant effects on the whiteness, WHC, gel strength, molecular force, and water distribution of MP gels. The WHC, gel strength, molecular force, and immobilized water content of each treatment group were higher than the control group, but the whiteness and free water content were lower than the control group.

## 4. Conclusions

This study evaluated the effects of different additions of SCF and ICF on the whiteness, gel strength, WHC, molecular force, rheology, water distribution, and microstructure of mutton MP gel. Treated with 2.5–10% SCF could significantly increase the G′ of MP gels and improve the viscoelasticity of thermally induced gels. Furthermore, SCF could strengthen the interaction between polysaccharide–protein or between protein–protein to form a relatively tight network structure, thereby improving the gel strength and WHC of MP gels. Compared with the control group and other treatment groups, the gel of the 5% SCF treatment group had the most ideal state. As an insoluble polysaccharide, the addition of 2.5–10% ICF could significantly increase the WHC and gel strength of MP gels. However, the thermally induced gel structure was relatively rough, uneven in structure, and contained plenty of pores. These data showed that SCF could improve the gel properties of mutton MP, and the effect was better than that of ICF. In conclusion, SCF can be used as a potential functional-enhancing ingredient in the meat industry.

## Figures and Tables

**Table 1:** Effect of different denaturants on the solubility of MP gels.

Sample	Solubility (%)		
	Urea	SDS	β-ME
Control	20.87 ± 0.08 ^b^	40.02 ± 0.62 ^d^	16.32 ± 0.19 ^c^
SCF + 2.5%	21.60 ± 0.37 ^ab^	44.62 ± 1.01 ^abc^	17.50 ± 0.61 ^bc^
SCF + 5%	22.60 ± 0.06 ^ab^	47.19 ± 0.34 ^a^	24.77 ± 0.90 ^a^
SCF + 7.5%	21.22 ± 0.76 ^ab^	44.69 ± 0.33 ^abc^	19.41 ± 1.71 ^bc^
SCF + 10%	21.84 ± 0.06 ^ab^	45.82 ± 0.16 ^ab^	20.71 ± 0.03 ^b^
ICF + 2.5%	20.22 ± 0.55 ^b^	42.56 ± 1.04 ^bcd^	16.32 ± 0.12 ^c^
ICF + 5%	20.75 ± 0.37 ^b^	42.13 ± 0.67 ^bcd^	22.67 ± 0.22 ^b^
ICF + 7.5%	21.54 ± 0.62 ^ab^	39.78 ± 1.13 ^d^	21.27 ± 0.44 ^b^
ICF + 10%	20.94 ± 0.59 ^ab^	41.54 ± 1.60 ^cd^	20.95 ± 0.34 ^b^

Each treatment was repeated at least in triplicate. Data are expressed as the mean ± SD. Different letters indicate significant differences (*p* < 0.05).

## Data Availability

The data presented in this study are available on request from the corresponding author.
